# Antimicrobials, Stress and Mutagenesis

**DOI:** 10.1371/journal.ppat.1004445

**Published:** 2014-10-09

**Authors:** Alexandro Rodríguez-Rojas, Olga Makarova, Jens Rolff

**Affiliations:** Evolutionary Biology, Institute for Biology, Free University Berlin, Berlin, Germany; Georgetown University, United States of America

## Abstract

Cationic antimicrobial peptides are ancient and ubiquitous immune effectors that multicellular organisms use to kill and police microbes whereas antibiotics are mostly employed by microorganisms. As antimicrobial peptides (AMPs) mostly target the cell wall, a microbial ‘Achilles heel’, it has been proposed that bacterial resistance evolution is very unlikely and hence AMPs are ancient ‘weapons’ of multicellular organisms. Here we provide a new hypothesis to explain the widespread distribution of AMPs amongst multicellular organism. Studying five antimicrobial peptides from vertebrates and insects, we show, using a classic Luria-Delbrück fluctuation assay, that cationic antimicrobial peptides (AMPs) do not increase bacterial mutation rates. Moreover, using rtPCR and disc diffusion assays we find that AMPs do not elicit SOS or *rpoS* bacterial stress pathways. This is in contrast to the main classes of antibiotics that elevate mutagenesis via eliciting the SOS and *rpoS* pathways. The notion of the ‘Achilles heel’ has been challenged by experimental selection for AMP-resistance, but our findings offer a new perspective on the evolutionary success of AMPs. Employing AMPs seems advantageous for multicellular organisms, as it does not fuel the adaptation of bacteria to their immune defenses. This has important consequences for our understanding of host-microbe interactions, the evolution of innate immune defenses, and also sheds new light on antimicrobial resistance evolution and the use of AMPs as drugs.

## Author Summary

Cationic antimicrobial peptides are ancient and ubiquitous immune effectors that multicellular organisms use to kill and police microbes, whilst antibiotics are mostly employed by microorganisms. Here we provide a new hypothesis to explain this widespread adoption of antimicrobial peptides. We show that cationic antimicrobial peptides (AMPs) do not increase bacterial mutagenesis, as they do not elicit bacterial stress pathways. Those stress pathways increase the mutation rate when bacteria are treated with antibiotics. Employing AMPs hence seems advantageous for multicellular organisms, as it does not fuel the adaptation of bacteria to their immune defenses. This has important consequences for our understanding of host-microbe interactions, the evolution of innate immune defenses, and also sheds new light on antimicrobial resistance evolution and the use of AMPs as drugs.

## Introduction

Ever since the advent of antibiotics, bacterial resistance has evolved and spread very rapidly [1] and recently has been shown to be ancient [2]. This led to the suggestion to use cationic antimicrobial peptides (AMPs) as alternatives, as these have been successfully used by multicellular organisms over millions of years [3]. While isolates from patients and wild animals display cationic antimicrobial peptide resistance [4], [5], which can be readily achieved *in vitro* [6], a fundamental observation still holds: cationic antimicrobial peptides are core components of the antimicrobial defences in animals [3], whereas synthesis and use of antibiotics is common in micro-organisms [7].

Here we suggest an explanation for this dichotomy based on the firmly established observation that antibiotics elicit stress-induced mutagenesis, which has been proposed to be adaptive in stressful environments as it increases evolvability [8], [9]. When stress leads to DNA damage, a common way of antibiotic action, the bacterial SOS pathway is activated and the DNA is repaired using an error-prone alternative polymerase [8]. The other main stress pathway *rpoS* increases mutation rates in a similar fashion [10]. It has been suggested that antibiotic treatment should be enhanced with mutation inhibitors interfering with the SOS pathway to increase the lifetime of antibiotics [11]. Antibiotics mostly interfere with replication, transcription and protein synthesis, in contrast cationic antimicrobial peptides of multicellular organisms mostly target the cell wall [12]. As such there is little potential of eliciting the SOS or *rpoS* stress pathways and to elevate bacterial mutation rates, a notion that we test here. This addresses the novel hypothesis that the use of antimicrobial peptides in multicellular organisms as a main effector against pathogens has been selected for because of the differences in mutagenesis between AMPs and antibiotics.

To investigate our hypothesis we selected a panel of AMPs and three conventional antibiotics as controls. We selected different cationic antimicrobial peptides such as Cecropin A and Melittin (both from insects) and magainin II and its derivative Pexiganan, LL-37 and a human lysozyme from vertebrates to represent different branches of the metazoa. For Mellitin, Magainin, Pexiganan and LL-37 the proposed killing mechanism is toroidal pore forming, while cecropin is considered to form a carpet on the bacterical cell wall [13]. As controls, we use Kanamycin (targets protein synthesis), Ampicillin (targets cell wall synthesis) and Ciprofloxacin (blocks topoisomerases) to represent the most important families of bactericidal antibiotics (Aminoglycosides, Beta-Lactam, Fluoroquinolone).

## Results

### Mutation rates of *E. coli* treated with antimicrobial peptides and antibiotics

We first investigated the mutation rates of *E. coli* using a Luria-Delbrück fluctuation test [14]. We used the *Escherichia coli* strain MG1655 and the concentration of all antimicrobials was adjusted to limit bacterial growth to 50% in four hours of treatment.

**Figure 1:**
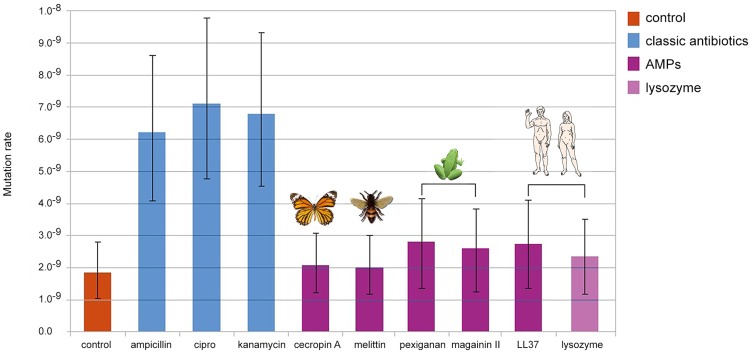
Changes in mutation rate in E. coli MG1655 induced by different antimicrobial treatments. Error bars show confidence interval for mutation rate estimation by plating in Rifampicin (100 µg/ml) and using the maximum likelihood method. Each bar represents the mutation rate from 10 independent cultures. All cultures were treated for 4 hours, with their corresponding MIC50 values for each antimicrobial as follow: 3.2 µg/ml of ampicillin, 0.05 µg/ml of ciprofloxacine, 1.6 µg/ml of kanamycin, 6 µg/ml of cecropin A, 12.8 µg/ml of human lysozyme, 8 µg/ml of LL-37, 1.6 µg/ml of melittin, 64 µg/ml of magainin II and 1.6 µg of pexiganan.

We found that bacteria, when treated with Ampicillin, Ciprofloxacin and Kanamycin, show 3 to 4 fold increase in mutation rate consistent with previous reports [15], [16]. By contrast, none of the groups treated with AMPs or human Lysozyme, showed changes in comparison to the control (Figure 1). The differences in mutation rates, as we observed between antibiotics and AMPs, are sufficient to promote the evolution of antibiotic resistance [17].

As the killing mechanisms differ between AMP and antibiotics we repeated the fluctuation assay using a gradient of sub-inhibitory to inhibitory concentrations of Mellitin and Pexiganan. The mutation rates did not change (Figure S1). Since the action of almost all cationic peptide is similar, attraction to the cell membrane by net charge [12], we assume that this result holds for many AMPs. It is also possible that the differences in mutation rates we found are caused by different dynamics in cell death [18] that may influence the number of cell divisions per culture. This is a general concern in all experiment estimating the mutation rate starting from different inoculum sizes [14]. To examine this possibility we estimated the mutation rate using culture with different inoculum sizes (10^2^, 10^4^, 10^6^, 10^8^ cfu/ml). In our settings, all conditions showed similar mutation rates (Figure S2) comparable to that of the control group in our first experiment (Figure 1).

### Stress pathway induction by antimicrobial peptides and antibiotics

SOS and *rpoS* mediated mutagenesis are well supported by empirical work [8], and it was recently proposed that antibiotics promote ROS formation and kill bacterial by lethal induction of hydroxyl-mediated DNA damage [15]. Here we explored this new and debated proposition [19] also for AMPs. We assessed AMP killing curves in *E. coli* MG1655 in the presence of the ROS scavenger thiourea (100 mM) and used Kanamycin as positive control. Consistent with the literature we found reduced sensitivity to Kanamycin when thiourea is present, the killing effect by antimicrobial peptides was insensitive to the presence of this ROS scavenger (Figure S3). AMP killing does not seem to depend on ROS levels.

**Figure 2:**
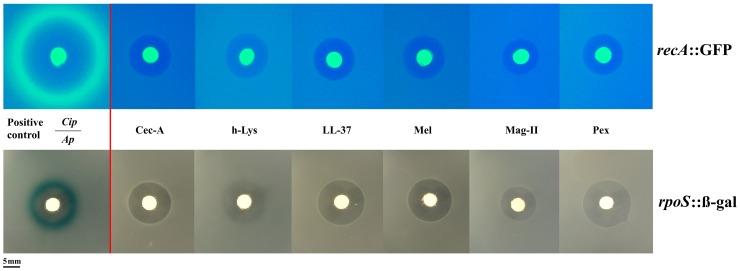
Antimicrobial peptides do not elicit the SOS (top panel) or the rpoS pathway (bottom panel). Induction of the SOSresponse or rpoS response is shown by a fluorescent ring around the zone of clearance (green for *recA*::GFP, blue for *rpoS*::β-gal at the edge of the inhibition zone). Positive controls with antibiotics (Cip: Ciprofloxacin, Ap: Ampicillin) are shown on the left. The bright central disc is a filter paper as source of the antibiotic/antimicrobial [22]. The amount of animicrobials on paper disks consisted of 50 µg of ampicillin, 5 µg of ciprofloxacine, 50 µg of cecropin A (Cec-A), 256 µg of human lysozyme (h-Lys), 50 µg of LL-37, 20 µg of Melittin, 200 µg of magainin II, and 20 µg of pexiganan.

To understand more deeply the difference in the bacterial response to AMPs and classic antibiotics, we used two approaches. Firstly, we used *E. coli* reporter strains for the two main stress pathways, *rpoS* and SOS in an agar diffusion assay. These two pathways have been shown to be involved in elevating mutation rate [8], [9]. Yet, AMPs did not activate these pathways (Figure 2) in contrast to the positive controls. As AMPs are known to attack mostly the cell membrane, we employed a suite of different stress pathway genes to assess their expression by qPCR.

We chose 12 stress-pathway genes, comprising components of the SOS, *rpoS* and envelope stress pathways (table S3) and examined their expression under pexiganan, melittin and lysozyme treatment: only pexiganan elicited an overexpression of three envelope stress genes (*marR*, *phoP* and *phoQ*) (table S3). None of the AMP treatments resulted in differential expression of genes related to stress-induced mutagenesis, which is consistent with very recently reported results on LL37 [20]. Despite these consistent results on a lack of stress-pathway induction the study on LL 37 [20] proposes elevated mutagenesis but does not report mutation rates (see Methods).

## Discussion

It is conceivable that the differences in mutation rates we report are an underestimate of the situation *in vivo*. In our experiment we did not study horizontal gene transfer. It is noteworthy that it has been shown before that alteration of the cell membrane, one of the primary resistance mechanisms of bacteria against AMPs [4], reduces cell competence [21]. By contrast, antibiotics eliciting the SOS response, might not only induce increased mutations via the DNA repair mechanisms but also lead to enhanced horizontal transfer [22].

Mutation rate is an important determinant of speed of adaptation in resistance evolution against AMPs and antibiotics [15], [23],[24]. Theoretical work strongly implies that adaptation to an antimicrobial in concentration gradients, as presumably encountered in most natural situations, can be greatly accelerated by increasing mutation rates through stress-induced mutagenesis [23]. In this model scenario faster killing, and AMPs tend to kill quicker than antibiotics [25], further reduces bacterial adaptation to antimicrobials. While our results are based on *E. coli* as a model and a particular panel of AMPs, we suggest, given the conserved nature of mutagenesis mechanisms in bacteria [8] and the conserved nature of AMPs in Metazoa [3] it is likely to be a more general phenomenon.

The fact that AMPs do not elevate mutation rates has, we propose, at least two more interesting implications. First, symbiont genome evolution can be accelerated by increased mutation rates [26] and mutation rate negatively is correlated with genome size in microbes [27]. Host-symbiont/commensal interactions can be mediated by AMPs [5] [28] but it remains to be seen if and how the host by employing AMPs does contribute to accelerated genome reduction in symbionts. Understanding the role of AMPs in bacterial mutagenesis will, we suggest, help to understand animals in a bacterial world [29]. One other interesting aspect is that the generation times of metazoans and their commensal, symbiotic or pathogenic bacteria often differ by orders of magnitude [30] resulting in within-host evolution of bacteria. The benefit of AMPs for the metazoan host can be considered to not further fuel the evolvability of the bacteria. By contrast, antimicrobials produced by bacteria are used in the context of interactions between organisms with very similar generation times.

Secondly, AMPs are used as antimicrobial drugs. *In vitro* experiments showed that adaptation to AMPs displays similar kinetics as antibiotic resistance evolution [31]. The situation *in vivo*, when a drug is used to support the overwhelmed immune system is almost certainly very different. The additional benefit of an unaltered mutation rate (including reduction in HGT) under AMP treatment contrasts with elevated mutation rates under antibiotic treatment. Changes in the mutation rate as reported here are in the range of mutation rates closely linked with disease emergence [32] and evolutionary rescue of populations of microorganisms [33].

## Materials and Methods

### Bacterial strains and growth conditions

*E. coli* K-12 MG1655 was used for all experiments, grown in liquid Mueller-Hilton (MH) agar or agarose, unless otherwise indicated. All experiments were carried out at 37°C.

### Preparation of reporters

The plasmid pUA66-P*recA*::GFP harbors a green fluorescent protein (GFP) transcriptional fusion with the *recA* promoter that is under control of lexA [34]. The plasmid was introduced into the electrocompetent cells of the strain MG1655 by electroporation. Transformed clones were selected on LB agar plates containing 30 µg/ml of kanamycin. The expression of GFP under SOS activation was determined by observing the fluorescence close to the inhibition area after addition of the SOS inducer mitomycin C.

A chromosome-integrated fusion reporter for *rpoS* with β-galactosidase, kindly provided by Regine Hengge (Humboldt University of Berlin, Germany), was transferred from the strain *E. coli* MC4100 via P1vir phage transduction to the *E. coli* strain MG1655 Δ*lacZ*. The reporter contains the hybrid protein RpoS742::LacZ (which carries the N-terminal 247 amino acids which include α2.5 of σS). This reporter preserves post-translational proteolytic control of σS expression [35].

The strain MG1655 Δ*lacZ* was obtained by disrupting the entire lacZ gene. Briefly, transformants carrying a red recombinase helper plasmid, pKD46, were grown in 5-ml SOB medium with ampicillin (100 µg/ml) and L-arabinose at 30°C to an OD600 of ≈0.6 and then made electrocompetent. PCR products with homology regions were gel-purified, digested with restriction enzyme *Dpn*I, column purified (MinElute PCR Purification Kit, Qiagen). Competent cells in 50 µl aliquots were electroporated with 100 ng of PCR product. Cells were added immediately 0.9 ml of SOC, incubated 1 h at 37°C, and then 100 µl aliquots spread onto LB agar with kanamycin (30 µg/ml), 40 ug/ml of 5-bromo-4-chloro-3-indolyl-3-D-galactoside (X-Gal) and 0.1 mM of isopropyl β-D-thiogalactopyranoside (IPTG). White colony resistant transformants were selected after 24 h of incubation. The correct inactivation of lacZ gene was verified by PCR.

The RpoS742::LacZ reporter construct was transferred from MC4100 to the strain *E. coli* MG1655 Δ*lacZ* following an improved protocol describe by Moore for P1 *vir* phage transduction with minor modifications. The transduced strain was selected on agar plates with M9 minimal medium containing 1% of lactose as only carbon source and supplemented with kanamycin (20 µg/ml) and 40 ug/ml of X-gal. A few blue transformants were regrown in MH with kanamycin (30 µg/ml). The phenotype of the new strain, namely MG1655 *rpoS*::*lacZ*, was verified by monitoring the β-galactosidase activity as described by Miller [33]. A correct phenotype corresponded with a growing β-galactosidase expression from exponential phase to stationary phase as σS does. Briefly, overnight cultures of MG1655 Δ*lacZ* (negative control) and its derivative containing the fusion reporter RpoS742::LacZ were diluted 1∶100 in 100 ml of LB. After 2 hours (OD_600_∼0.5), the pellets of six sequential aliquots of 1 ml, that were taken at intervals of 1 hour (from the mid exponential phase to stationary phase) were re-suspended in 1 ml of Z-buffer (NA_2_HPO_4_ 16.1 g/L, NaH_2_PO_4_ 5.5 g/L, KCl 0.75 g/L, MgSO4 0.246 g/L and β-mercaptoethanol 0.3% (v/v). Bacteria were lysed with 0.1% SDS and 20 µl of chloroform. The reaction was started by adding 200 µl of ONPG (4 g/ml) per tube, and it was stopped by the addition of 500 µl of 1 M NaCO_3_ solution (pH 9). The experiment was done at room temperature. β-galactosidase activity was expressed as Miller units.

### Soft-agarose transcription fusion reporter assay for *rpoS* and *recA* reporter assay

All antibiotics were purchased from Carl Roth GmbH, pexiganan was kindly provided by M. Zasloff (Georgetown University), melittin was purchased from Serva, and human lysozyme was obtained from Sigma. To qualitatively assess antimicrobial-mediated induction of both stress pathways, overnight cultures of the *recA* (SOS pathway) and and *rpoS* (general stress response σS) reporters, were diluted 1 in 100 in fresh MH and cultivated with shaking to mid exponential phase (OD_600_∼0.5). Aliquots of 2.5 ml of culture were mixed in equal part with MH containing 0.6% of pre-cooled agarose (to a final concentration of 0.3% of agarose) at 40°C and were poured immediately onto MH agarose plate at 1.5%. In the case of MG1655 *rpoS*::*lacZ* strain, the plates also contained X-gal to a final concentration of 40 µg/ml, and 80 µg/ml for the pre-cooled agarose. Antimicrobial-containing filter discs of Watman No. 1 (5 mm Ø) were deposited onto the middle of agarose plates and were incubated during 6 and 24 hours before visualization at 37°C. Discs with ciprofloxacin (5 µg) and ampicillin (10 µg) were used as positive control to assess the induction of *recA* and *rpoS* respectively. The others disks contained different concentrations of antimicrobial peptides to obtain a similar inhibition area: 50 µg of cecropin A (Cec-A), 256 µg of human lysozyme (h-Lys), 50 µg of LL-37, 20 µg of Melittin, 200 µg of magainin II, and 20 µg of pexiganan. The plates carrying *recA* reporter were observed using a blue light transiluminator (Biosteps GmbH, Germany) for excitation of the GFP and a filter (502–538 nm) to visualize the emission of fluorescence, while the plates with MG1655 *rpoS*::*lacZ* reporter were observed under natural light for direct visualization of a blue ring of X-gal hydrolysis surrounding inhibition area, in case of induction. All assays were repeated at least three times.

### Minimal inhibitory concentration (MIC)

MICs were determined according to CLSI recommendations by a micro-dilution method with the exception that the inoculum size that was adjusted to 2×10^8^ cfu/ml from a regrowth of overnight cultures to be consistent with the mutagenesis experiments. The MIC was defined as the antimicrobial concentration that inhibited growth after 24 h of incubation in liquid MH medium at 37°C. Polypropylene non-binding plates (Th. Geyer, Germany) were used for all experiments.

### Determination of MIC50

The MIC50s for all antimicrobials were determined by inoculating strains grown to mid-log phase into the wells of a 96-microwell plate. Approximately 10^2^ cells from overnight cultures were inoculated into tubes containing 10 ml of Mueller Hinton Broth medium, and the tubes were incubated at 37°C with strong shaking until the mid-log phase of growth (approximately 10^8^ cells/ml). Then, 100 µl of 2–3×10^8^ cells from these cultures were inoculated into each well containing 100 µl of Mueller Hinton Broth and starting from the MIC concentrations were serially diluted by a factor of 2 (lowest concentration 1/128 of the MIC) for all antimcirobials. The plates were incubated at 37°C for 4 h with continuous shaking in a plate reader (Synergy 2, BioTeK). After four hours, aliquots from wells with OD^600^ readings ranging from 10 to 90% values in comparison with the control, were serially diluted in sterile 0.9% NaCl, and incubated during 1 hour to allow the resolution of filaments as consequence of SOS activation, if any. Bacterial suspensions were plated in Mueller Hinton Broth agar plates to estimate the viability as colony forming units (CFUs). Five replicates per concentration were prepared and the experiments were repeated twice. MIC50s at 4 hours were defined as the concentrations at which 50% of growing reduction in terms of CFUs in comparison to the control were observed.

### Mutagenesis experiments

To understand mutagenesis the most important parameter to estimate is the mutation rate using a fluctuation assay [14], other possible correlates of mutagenesis have severe shortcomings (for example mutant frequencies as reported in a recent study on LL37 [20], a detailed description of the methodological problems is available on request).

In order to obtain comparable results from the activity of classic antibiotics and AMPs, the mutation rate experiments were performed as follows. The duration of treatment was set to 4 hours for all antimicrobials, and the concentrations were adjusted to obtain around 50% of inhibition of grow in comparison to the a non-treated control. Three ml of exponentially growing culture of *E. coli* MG1655 (∼2×10^8^ cells/ml) were centrifuged at 4000 g for 10 minutes and re-suspended in the same volume of MH with different concentrations of antimicrobials at 37°C with shaking (200 rpm). After incubation, the cultures were centrifuged for 10 min at 4000 g. The pellet was re-suspended, washed twice with MH and re-suspended in 3 ml of fresh medium and incubated overnight (16 h) at 37°C with shaking. This step is necessary to resolve the filaments that some antimicrobials induce due to their toxic action and allow recovery of cells under stress. Cultures were diluted and plated to determine the number of colony forming units in MH agar plates. The number of mutants was estimated by the number of colonies growing on rifampicin (100 µg/ml). Mutation rates were calculated by maximum verisimilitude method and data were processed using the on-line web-tool for mutation rate determination Falcor (http://www.mitochondria.org/protocols/FALCOR.html). Every experiment consisted of ten independent cultures. The whole experiment was repeated twice.

We also assessed if different AMP concentrations (Fig. S1) or different inoculum sizes (10^2^, 10^4^, 10^6^, 10^8^ cfu/ml) influenced mutation rates (Fig. S2).

### Killing by AMPs under thiourea ROS scavenger

For two antimicrobial peptides, melittin and pexiganan, we assayed their respective MIC values in the presence or absence of the protective concentration of 100 mM of thiourea. Kanamycin was used a positive control. The procedure was the same as described in this work elsewhere for MIC50 (Fig. S3).

### Quantification of gene expression, sample acquisition

*Escherichia coli* strain MG1655 was used to assess the response of twelve stress pathways-related genes to three antibiotics (kanamycin, ciprofloxacin and ampicillin) and two antimicrobial peptides (AMPs) (pexiganan, melittin) and human lysozyme in comparison with a non-treated control. Preparation of each of the six treatments and the control samples was carried out by two operators on three consecutive days, thereby comprising three biological replicates for each of the six experimental and one control groups. To obtain the starting material for the experiment, a fresh bacterial culture was grown from a glycerol stock on Mueller-Hinton (MH) agar plates at 37°C for 18–24 hours. One well-isolated colony per biological replicate was used to inoculate 2 ml of liquid MH (Sigma) in a 10 ml sterile polypropylene tube and incubated at 37°C for 16 hours with mild shaking. The overnight (ON) culture was then diluted 1∶100 in MH, subdivided into seven 10 ml aliquots and incubated in 50 ml-Falcon tubes at 37°C for 2.5 hours at 220 rpm. When the OD_600_ of bacterial cultures reached 0.5–0.7, respective antibiotics and AMPs were added to a concentration to match with those used in the mutagenesis experiments. These bacterial cultures were incubated for 20 minutes at 37°C with gentle shaking. Two ml of bacterial cultures from each treatment and the control were collected, immediately centrifuged at 10000 *g* for 2 minutes and the supernatant was removed to stop the treatment. The bacterial pellet was then resuspended in 1 ml MH, followed by an addition of 1 ml of RNAprotect Bacteria Reagent (Qiagen), 5 minutes incubation and centrifugation at 10000 *g* for 2 minutes at room temperature. The supernatant was discarded and the bacterial pellet was immediately frozen and stored at −80°C until RNA extraction.

### Quantification of gene expression, RNA isolation

Total RNA was isolated using RNeasy kit (Qiagen) according to the manufacturer's instructions and eluted in 50 µl of RNase-free water. The nucleic acid yield and purity were determined by measuring the optical density at A260/280 using a Nanodrop spectrophotometer (Thermo Scientific). RNA samples were treated with TURBO DNase (Life Technologies). Briefly, ten µg of RNA were used in a total volume of 500 µl containing 20 units of TURBO DNase, incubated for 30 minutes at 37°C, immediately followed by RNeasy (Qiagen) cleanup and elution in 30 µl of RNase-free water. Following DNase treatment, RNA integrity was assessed using Agilent RNA 6000 Nano kit and 2100 Bioanalyzer instrument (both Agilent Technologies). All samples had RIN values above 8.

### Quantification of gene expression, cDNA synthesis

High-Capacity cDNA Reverse Transcription Kit with RNase Inhibitor (Applied Biosystems) was used for cDNA synthesis. Initially, to ensure linear conversion of the transcripts, the dynamic range of reverse transcription reaction was tested by performing a standard curve with cDNA, synthesised using various input amounts of pooled RNA. Total RNA (250 ng per reaction) and random primers were used for cDNA synthesis. To obtain sufficient amount of cDNA, several batches of 20 µl RT reactions were pooled, diluted 50-fold with RNase-free water and stored in single-use aliquots at −80°C until further use. All 21 samples were tested for presence of contaminating genomic DNA by running the *mdoG* assay with cDNA and the respective no reverse transcription (−RT) controls. There was no amplification in the majority of −RT controls. In −RT samples with detectable amplification, difference in Ct values when compared with +RT varied between the samples, but was no less than 10 cycles for all of them with the lowest Ct values in −RT control samples ≥30.

### Primer design

*Escherichia coli* strain K-12 substrain MG1655 complete genome (accession U00096) sequence was downloaded from NCBI database (www.ncbi.nlm.nih.gov) and used for primer design. Target sequence accession number, location of the amplicon, amplicon length and primer sequences for each assay can be found in table S2. Primers were designed using Primer Express software (Applied Biosystems) and optimized for annealing temperature of 60°C. Each primer pair and amplicon were checked for secondary structure formation using Oligo Tool (Integrated DNA technologies, http://eu.idtdna.com/analyzer/Applications/OligoAnalyzer/). Primer and amplicon specificities were first tested *in silico* by performing a BLAST search against the complete genome. Primers were synthesised by Metabion GmbH (Germany) and purified by desalting.

### Quantification of gene expression, quantitative real-time PCR

PCR reactions were prepared manually in a total volume of 10 µl by mixing 5 µl 2× KAPA SYBR FAST ABI Prism master mix (KAPA Biosystems), 0.2 µl forward and reverse primer mix (10 µM each primer), 2.8 µl RNase-free water and 2 µl cDNA in MicroAmp Fast Optical 48-wells reaction plates (Applied Biosystems). PCR reactions were run in the Fast mode using StepOne thermocycler (Applied Biosystems) with the following cycling conditions: 95°C 3′/40×(95°C 3″/60°C 20″)/melting curve analysis. Each assay was run in duplicate. No template controls (NTC) were run with each assay. Presence of a single specific product was verified by running a melt curve analysis followed by visualisation of the qPCR product in a 2% agarose gel stained with SYBR Safe DNA Gel stain (Life Technologies). qPCR amplicons of each gene were cloned into the pGEM-T vector (Promega) following the instructions manual and subsequently sequenced to confirm the specificity of the assays. Additionally, each PCR assay was tested for reaction efficiency as follows: equi-molar amounts of cDNA from all 21 samples were pooled together and used for the preparation of the standard curve by serial dilution (1∶3) of pooled cDNA over five dilution points and run in triplicate.

### Quantification of gene expression, data analysis

Amplification curves were first visually examined in the StepOne software (Applied Biosystems). No baseline and threshold line adjustments were necessary. Ct values of the technical replicates were averaged and used for relative gene expression analysis in the REST 2009 software (Qiagen). Expression of target genes was normalized to the expression levels of three reference genes (*arcA*, *mdoG* and *tus*), selected based on the assessment of the expression stability across all experimental conditions using BestKeeper software [36]. Reaction efficiency information inferred from the standard curve data was used to correct for differences in amplification efficiencies in the REST 2009 software. Default settings (2000 iterations) were used for randomisation and bootstrapping analysis to test significance of gene expression. Expression values with *p*-values ≤0.05 were assigned as differentially expressed. The relative gene expression results can be found in the supplementary Table.

### Quantification of gene expression, MIQE standards

The Minimum Information for Publication of Quantitative Real-Time PCR experiments (MIQE) guidelines-compliant checklist were fulfilled.

## Supporting Information

Figure S1 **Mutation rates of** ***E. coli*** **MG1655 from cultures with different concentrations of antimicrobial peptides.** The cultures were treated with melittin and pexiganan with concentrations ranging from 0.5 to 4 µg/ml. Note that higher concentrations cannot be assayed because they are lethal and as AMPs kill much quicker than antibiotics the cultures do not recovered from the treatment. Error bars show confidence interval for mutation rate estimation by plating in Rifampicin (100 µg/ml) and using the maximum likelihood method.
(PDF)

Figure S2 **Mutation rates of** ***E. coli*** **MG1655 from cultures with different inoculum sizes.** Error bars represent 95% of confidence intervals. Error bars show confidence interval for mutation rate estimation by plating in Rifampicin (100 µg/ml) and using the maximum likelihood method.
(PDF)

Figure S3 **Killing curves of AMPs and kanamycin in the presence of 100 mM of thiourea.**
(PDF)

Table S1 **MIC and MIC_50_ (4 hours) values for** ***E. coli*** **MG1655 for different antibiotics and antimicrobial peptides.**
(PDF)

Table S2 **Primers used for relative gene expression quantification by real time PCR.**
(PDF)

Table S3 **Relative gene expression for** ***E. coli*** **stress response genes in treatments with AMPs and antibiotics vs. non-treated bacteria.**
(XLS)
