# Supplementary figures and images for: Antimicrobials, Stress and Mutagenesis
Source: PLoS Pathog. 2014 Oct 9;10(10):e1004445. doi: 10.1371/journal.ppat.1004445 (PMC4192597; doi:10.1371/journal.ppat.1004445)

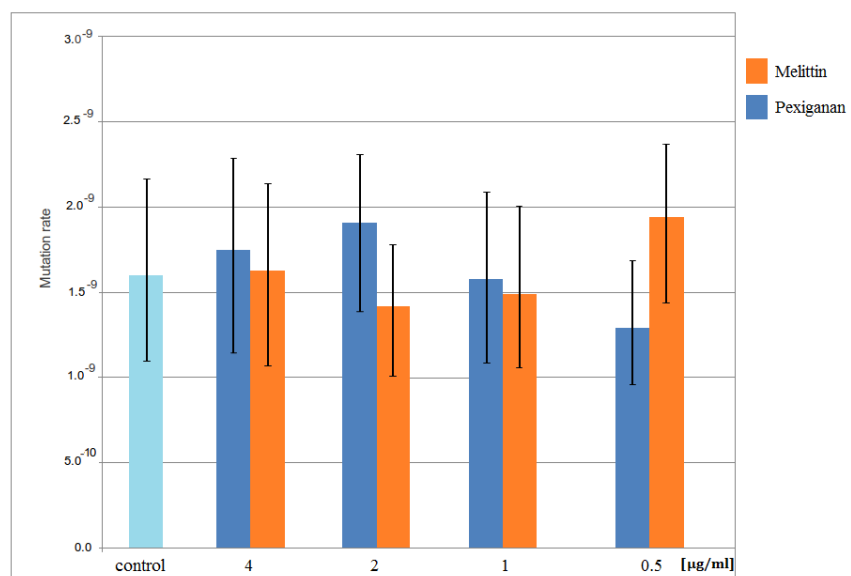

**Figure S1. Rojas et al.**

Supplement: Figure S1 — Mutation rates of E. coli MG1655 from cultures with different concentrations of antimicrobial peptides. The cultures were treated with melittin and pexiganan with concentrations ranging from 0.5 to 4 µg/ml. Note that higher concentrations cannot be assayed because they are lethal and as AMPs kill much quicker than antibiotics the cultures do not recovered from the treatment. Error bars show confidence interval for mutation rate estimation by plating in Rifampicin (100 µg/ml) and using the maximum likelihood method. (PDF) [file ppat.1004445.s001.pdf]

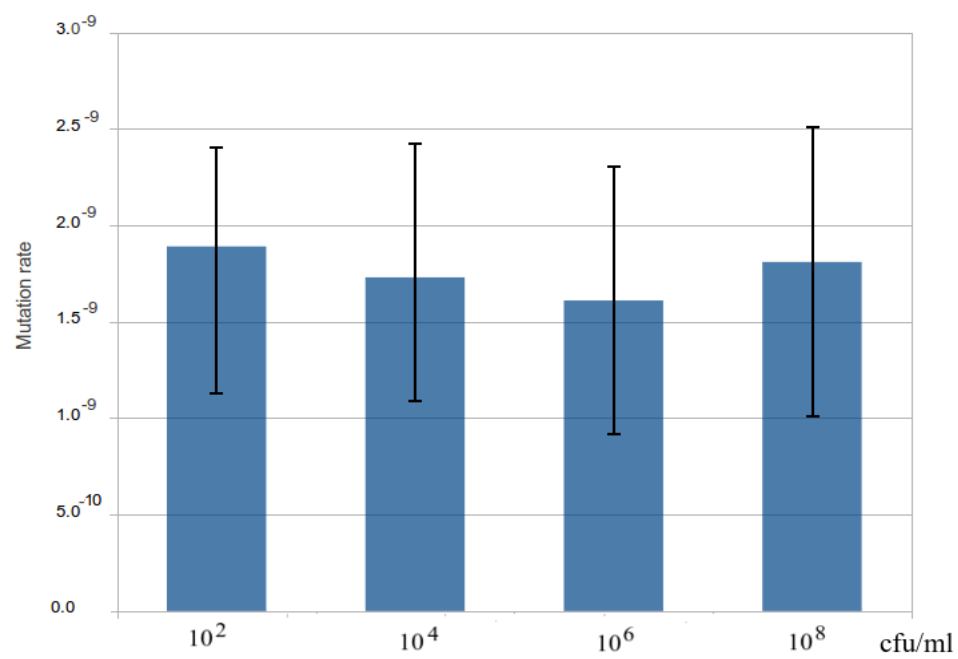

**Figure S2. Rojas et al**

Supplement: Figure S2 — Mutation rates of E. coli MG1655 from cultures with different inoculum sizes. Error bars represent 95% of confidence intervals. Error bars show confidence interval for mutation rate estimation by plating in Rifampicin (100 µg/ml) and using the maximum likelihood method. (PDF) [file ppat.1004445.s002.pdf]

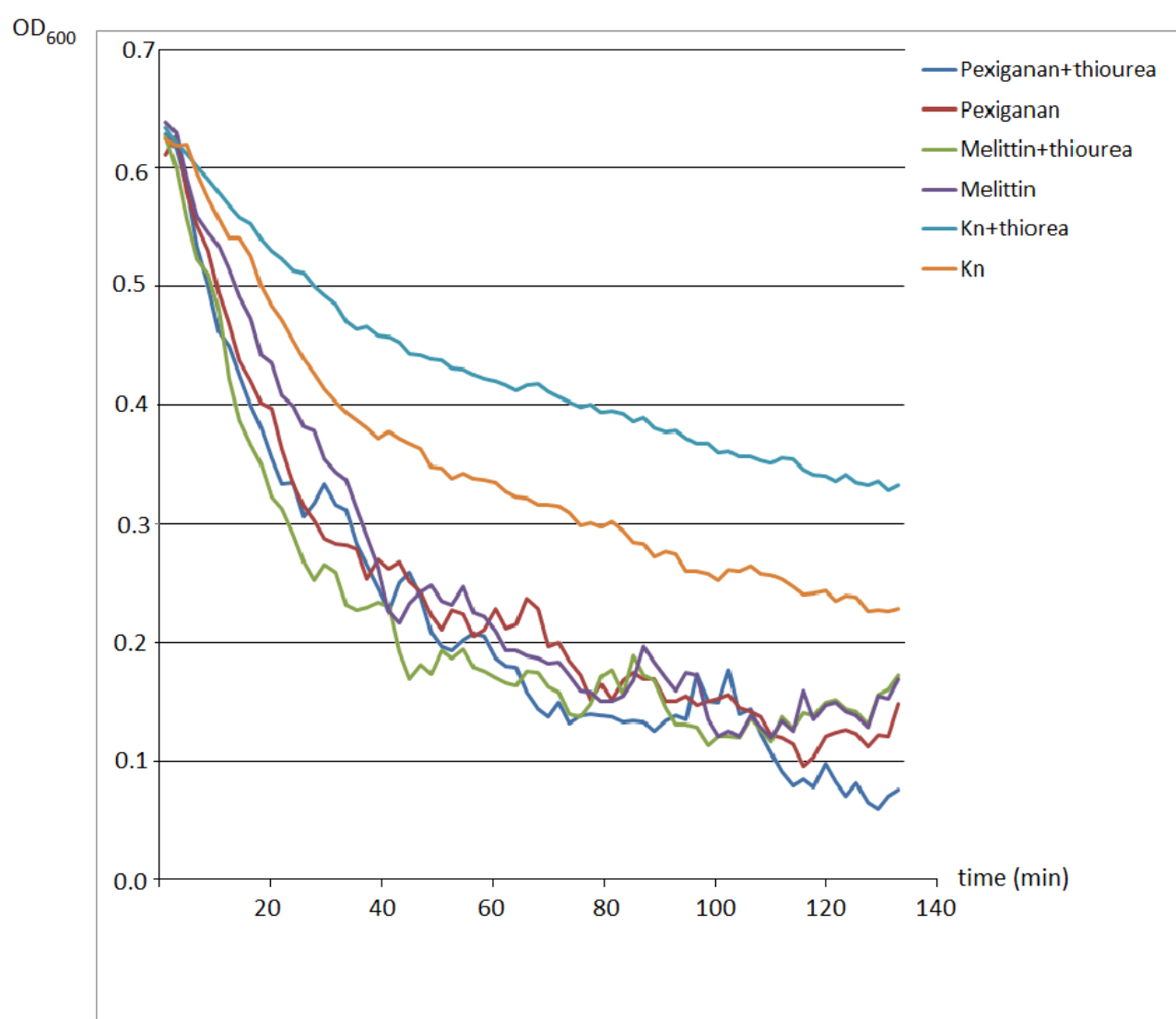

**Figure S3**

Supplement: Figure S3 — Killing curves of AMPs and kanamycin in the presence of 100 mM of thiourea. (PDF) [file ppat.1004445.s003.pdf]
