# Supplementary material for: Antimicrobials, Stress and Mutagenesis
Source: PLoS Pathog. 2014 Oct 9;10(10):e1004445. doi: 10.1371/journal.ppat.1004445 (PMC4192597; doi:10.1371/journal.ppat.1004445)
Supplement: Table S1 — MIC and MIC50 (4 hours) values for E. coli MG1655 for different antibiotics and antimicrobial peptides. (PDF) [file ppat.1004445.s004.pdf]

Table S1.

Antibiotic/AMPs	MIC (µg/ml)	MIC_{50} (µg/ml)
Ampicillin	4	3.2
Ciprofloxacin	0.125	0.05
Kanamycin	2	1.6
Cecropin A	8	6
Human lysozyme	20	12.8
LL-37	10	8
Melittin	2	1.6
Magainin II	100	64
Pexiganan	2	1.6
