# Supplementary material for: Antimicrobials, Stress and Mutagenesis
Source: PLoS Pathog. 2014 Oct 9;10(10):e1004445. doi: 10.1371/journal.ppat.1004445 (PMC4192597; doi:10.1371/journal.ppat.1004445)
Supplement: Table S2 — Primers used for relative gene expression quantification by real time PCR. (PDF) [file ppat.1004445.s005.pdf]

Table S2.

Gene	Assay	EcoGene Accession Number	Amplicon coordinates within the gene sequence, bp	Amplicon size, bp	Forward primer	Reverse primer
arcA	reference	EG10061	417-481	65	CAATGGTTGGGAACTGGACAT	GCTTGTACTGCTCGCCATCA
cpxA	target	EG10163	935-1001	67	AGTTGTGGAGTGAAGTGCTGGAT	ACTGTCAACGACTTGCCCATTT
cpxR	target	EG10020	561-617	60	CAAACGCCTGACGCCTTT	CGACGCAGGTTGGAAATGT
dinB	target	EG13141	719-701	61	GCATTTTGTGGGAGCGTAGTC	CGCAACCGTTCGCTGTTAA
lexA	target	EG10533	378-375	66	TTCAAGCCGAATGCTGATTTC	CATAATGCCGATATCTTTCATCGA
marR	target	EG11435	77-150	74	ATCGCCTGCTTAACGAGTATCTGT	GCGGATAGAGCAGAGCACCTT
mdoG	reference	EG11885	299-357	59	CCACCGCAGTCAAACGAAT	CTGAACATCGCCGAAAGTGA
oxyR	target	EG10681	83-140	58	GCCAGCCGACGCTTAGC	AACATCACGCCCAGCTCATC
phoP	target	EG10731	171-229	59	GGACGGTCTGTCACTGATTCG	CCAGAATCGGCAGTGAAACAT
phoQ	target	EG10732	1021-1079	59	GCCCCACTGCTGGACAAT	ACCCCTTTGCGTTGATACACTT
recA	target	EG10823	353-331	61	ACGCGCTGGACCCAATC	GAGCACAGCAGGTTGTCGATATC
rpoE	target	EG11897	216-273	58	ATGGCTGTATCGGATTGCTGTAA	ACGCCCCTGAGCAACCA
rpoS	target	EG10510	456-519	64	GACGATTGAACGGGCGATTA	CTTTACGATGTGAATCGGCAAAC
tus	reference	EG11038	495-563	69	CGCCACTTTACGCTTTGGTT	GCCAGGACTTCATCACGATGT
umuC	target	EG11056	193-254	62	TGTGGCGTGGTTTGCTTTAG	ATCACCCGATTGCTCATGTCT
